# Stakeholder perspectives on biosecurity adoption in the Colorado cattle supply chain: lessons from foot-and-mouth disease preparedness

**DOI:** 10.3389/fvets.2026.1937939

**Published:** 2026-10-02

**Authors:** Tais C. De Menezes, Emilia Ravetta, Rachel E. Lahoff, Kate J. Oviatt, John Ritten, Jason Lombard, Franklyn Garry

**Affiliations:** 1 Department of Agricultural and Resource Economics, Colorado State University, Fort Collins, CO, United States; 2 Sociology Department, Colorado State University, Fort Collins, CO, United States; 3 The Institute for Research in the Social Sciences, Colorado State University, Fort Collins, CO, United States; 4 AgNext, Colorado State University, Fort Collins, CO, United States; 5 Department of Clinical Sciences, Colorado State University, Fort Collins, CO, United States

**Keywords:** biosecurity, cattle, foot-and-mouth disease, preparedness, stakeholder engagement

## Abstract

Biosecurity is essential to protecting cattle health and maintaining continuity of business during high-consequence disease events, yet adoption of preparedness measures remains uneven across the beef supply chain. Using foot-and-mouth disease (FMD) as a motivating case, we examine how Colorado cattle-industry stakeholders define practical biosecurity and what lessons support preparedness before an emergency. Data were collected through 12 focus groups and 18 individual interviews with 75 stakeholders across nine cattle-industry sectors, followed by four cross-sector workshops with 56 participants. FMD was widely perceived as a distant, low-priority threat, while time, labor, cost, operational scale, traceability burdens, seasonal and geographic constraints, and limited veterinary access shaped what stakeholders considered feasible. Workshop participants identified visitor/vendor hygiene and controlled access as the most feasible measures, while quarantining new or returning animals received the most votes for both impact and urgency, followed by avoiding commingling with animals from other herds (impact) and knowing or asking about disease status of source herds (urgency). Trusted local messengers and cross-sector communication protocols emerged as central to practical, voluntary biosecurity improvement in Colorado. Strengthening biosecurity will require practical tools, trusted communication networks, and cross-sector planning that reflect the day-to-day realities of cattle operations.

## 1 Introduction

Biosecurity is central to protecting animal health, maintaining livestock production, and reducing the economic and social disruption associated with infectious animal diseases. In cattle production systems, biosecurity is not merely a set of technical recommendations implemented on individual premises; rather, it depends on the sustained participation of many actors whose decisions are shaped by operational constraints, economic incentives, trust, communication, and the structure of the supply chain (1–3). These dynamics are especially pronounced in the beef sector, where cattle move through multiple, weakly connected stages (cow-calf and stocker operations, feedlots, sale barns, and processing facilities), with veterinarians, extension agents, and regulatory agencies playing important supporting roles.

Foot-and-mouth disease (FMD) illustrates why preparedness matters. FMD is a highly contagious viral disease of cloven-hoofed animals and one of the most economically consequential transboundary animal diseases worldwide (4). Although the United States has been free of FMD since 1929, an incursion would trigger movement restrictions, disrupt domestic supply chains, cost export markets, and generate severe economic consequences across the livestock sector (5). Therefore, effective protection cannot rely on emergency response alone; by the time an outbreak is recognized, the ability to limit spread and maintain continuity of business depends on whether operations have already adopted feasible practices, developed plans, maintained records, and established trusted channels of communication (6).

The Secure Beef Supply (SBS) Plan embodies this preparedness logic, offering voluntary continuity-of-business guidance to support permitted movement of animals and products from premises with no evidence of infection during an FMD outbreak, particularly within a regulatory Control Area (7). Many of the SBS Plan’s core elements, such as obtaining a premises identification number, developing a written enhanced biosecurity plan, training personnel to monitor for clinical signs, and maintaining movement records, require advance preparation, reframing preparedness as a challenge of voluntary, sustained behavior rather than emergency logistics alone. Such longer-term commitments are often harder to sustain than short-term, outbreak-specific actions (e.g., restricting animal or product movement or intensifying cleaning and disinfection) because their benefits are uncertain, delayed, or largely invisible in the absence of an outbreak (3, 8, 9).

Understanding how stakeholders perceive these measures is therefore essential for designing strategies that are technically sound, practical, trusted, and likely to be adopted. The literature identifies risk perception, economic considerations, trust, social norms, and communication as recurring influences on adoption (1–3, 10), and recent work suggests that collaborative, two-way approaches may encourage more durable behavior change than top-down regulation (11, 12). In the U.S. beef sector, however, adoption of key preparedness practices remains limited and feasibility varies across production types, operation sizes, geographies, and market roles (13–15). Much of this U.S. evidence is quantitative and focused on single states or sectors; far less is known about how perceptions and the practical feasibility of recommended practices differ across the interconnected sectors of a regional supply chain.

This brief research report addresses this gap using qualitative data from stakeholder discussions and workshops across Colorado’s beef supply chain. FMD served as the concrete scenario grounding these discussions, but we treat it as an example of a broader biosecurity preparedness challenge. The guiding question is: what practical lessons from this work can help Colorado stakeholders strengthen biosecurity before an emergency occurs? Specifically, we examine how stakeholders across the supply chain perceive the feasibility of recommended practices, what barriers limit adoption, what incentives or tools could support sustained engagement, and how trusted communication and cross-sector coordination could strengthen preparedness across the chain.

## 2 Methods

### 2.1 Study design and participants

This study aimed to characterize current limitations and barriers to biosecurity adoption and to identify incentives and cross-sector coordination strategies that could increase voluntary adoption of enhanced biosecurity in the context of FMD. We used a qualitative, stakeholder-engaged design with two phases: (i) sector-specific focus groups and interviews to identify perceived barriers, facilitators, and incentives related to FMD preparedness and biosecurity adoption; and (ii) cross-sector workshops that translated these findings into practical priorities and potential action steps.

Between November 2024 and July 2025, the research team conducted 12 focus groups and 18 individual interviews across Colorado with 75 cattle-industry participants representing cow-calf producers, stocker operators, dairy producers, feedlots, processing/packing facilities, sale barns, brand inspectors, veterinarians, and Extension (Table 1). The research team used purposive recruitment to obtain representation across stakeholder groups, supplemented by convenience recruitment through team contacts and snowball recruitment through referrals. Eligibility required participants to be at least 18 years of age and to have current professional involvement in Colorado’s cattle industry. For the purposes of recruitment, professional involvement included ownership, management, employment, professional service, Extension, inspection, processing, marketing, or other direct involvement in cattle production or associated supply-chain activities. No minimum operation size or number of years of industry experience was required.

**Table 1:** Phase 1 focus group and interview participants by sector (*N* = 75).

Sector	Participants (n)
Cow-calf producers	26
Extension	10
Brand Inspectors	8
Veterinarians	6
Feedlot	6
Stockers	5
Processing/packing facilities	5
Sale Barns	5
Dairy Producers	4
Total	75

We then conducted four stakeholder workshops in 2026, held in Sterling (January 27th), Pueblo (February 9th), Fort Collins/Loveland (February 16th), and Grand Junction (March 11th), with 56 participants (Table 2). The workshops were conducted after completion and preliminary analysis of the phase 1 focus groups and interviews so that the themes identified in phase 1 could inform the workshop exercises and cross-sector prioritization. Workshop participants represented the sectors included in the focus groups and interviews, as well as additional organizations relevant to outbreak preparedness (county government, the Colorado Department of Agriculture, the Colorado Cattlemen’s Association, and a community animal response team), supporting cross-sector discussion of shared priorities. Because some participants represented more than one sector, sector counts in Table 2 sum to more than the 56 unique attendees. Of the 56 workshop participants, 21 had previously participated in a Phase 1 focus group or interview; therefore, the study included 110 unique participants across both phases.

**Table 2:** Sector representation among Phase 2 workshop participants.

Sector	Participants (n)
Cow-calf producers	27
Extension agents	14
Stockers	10
Veterinarians	10
Dairy producers	7
Feedlot operators	6
Brand inspectors	4
County government	4
Meatpackers	2
Colorado Cattlemen’s Association	2
Colorado Department of Agriculture	1
Community animal response team (CART)	1

Sector counts sum to more than 56 because some participants represented multiple sectors.

### 2.2 Data collection

Focus groups and interviews followed a semi-structured guide covering disease risk perception, current biosecurity practices, awareness of FMD preparedness resources, implementation barriers, incentives, communication needs, trusted messengers, and trust in institutional response. The same core semi-structured discussion guide was used across Phase 1 focus groups and individual interviews. Core questions and introductory information were standardized across sectors, while facilitators could use neutral follow-up probes to clarify sector-specific experiences or examples. The complete discussion domains and questions are provided in the Supplementary material. Facilitators introduced selected Secure Beef Supply Plan strategies as discussion prompts but did not endorse particular practices or present a preferred response. FMD grounded the discussions, but participants were also encouraged to discuss everyday biosecurity practices and the realities shaping their adoption. Focus groups were organized by sector. Interviews were used when scheduling, geography, availability, or role sensitivity (for example, when a participant’s professional or institutional position could make candid discussion of reporting, trust, or institutional response uncomfortable in a group setting) made one-on-one discussion more appropriate. Focus groups were designed to last approximately 2 h and individual interviews were designed to last approximately 30 min. Participants provided informed consent; the study was reviewed, approved, and determined exempt by the Colorado State University Institutional Review Board, protocol number 4502. Sessions were audio-recorded and transcribed verbatim, as well as documented through detailed notes.

The 2026 workshops centered on two structured exercises. In the first exercise, participants reviewed a list of biosecurity practices, added practices they believed were missing, and voted on the resulting set against three criteria: impact, feasibility, and urgency. The complete predefined list is provided in the Supplementary material. Voting was conducted with color-coded adhesive dots: each participant received nine dots (three per criterion) and placed them individually on the listed practices, with the option to apply multiple criteria to the same practice. The predefined list of nine practices was identical at all four workshops; practices added by participants could receive votes only at the workshop where they were proposed. Workshop attendance was 10 in Sterling, 16 in Pueblo, 17 in Fort Collins/Loveland, and 13 in Grand Junction. Across the four workshops, participants cast 158 impact votes, 149 feasibility votes, and 136 urgency votes (maximum possible: 168 per criterion). Participants then discussed how these varied by operation type and sector. The second activity, a Critical Shifts exercise adapted from the Tamarack Institute (16), asked participants to describe the current state of biosecurity, highlighting barriers to biosecurity adoption. Participants then described the opposing desired future state of biosecurity adoption using neutral, non-blaming language. Participants then prioritized shifts by their potential to advance biosecurity, enable other changes, require cross-sector collaboration, and generate stakeholder adherence. Participants then discussed realistic steps that could be taken within the next year, who would need to be involved, and what roles different sectors would play. Two research team members with training and experience in qualitative and participatory methods, including the Critical Shifts tool, designed and facilitated the workshops. Additional team members assisted with note-taking, logistics, and small-group support. Workshop outputs included voting results from the prioritization exercise, audio recordings of group discussions, group notes, large-group discussion notes, and written critical-shift statements. These materials were used to identify stakeholder-generated priorities and practical next steps for improving biosecurity across Colorado’s cattle industry. Participants provided informed consent. The research team submitted an amendment to the original IRB protocol (#4502) to include the workshops. Reflecting the different nature of the activities in the two phases, the amendment received a separate determination: the Colorado State University Institutional Review Board reviewed it and determined the workshop activities to be Not Human Subjects Research.

### 2.3 Data analysis

Focus group and interview transcripts were analyzed thematically in Dedoose using combined deductive and inductive coding. Deductive codes were derived from the complete set of discussion-guide domains and project core areas of interest (e.g., disease risk perception, biosecurity practices, barriers, incentives, communication, trusted messengers, traceability, veterinary access, and trust). The complete discussion guide and topic list are provided in the Supplementary material. Inductive themes and subthemes were subsequently developed through iterative review of the transcripts to capture recurrent ideas, sector-specific concerns, and unexpected themes raised by participants. Transcripts were coded by one member of the research team. Following an initial round of coding, a second researcher reviewed the coded data to compare coding strategies and the application of codes across the dataset. Differences were discussed and resolved through consensus, rather than through formal calculation of inter-coder reliability, and the coding framework was refined as needed to ensure that codes were applied consistently and reflected the data. Coded excerpts were then reviewed within and across sectors to identify shared patterns, divergent perspectives, and sector-specific differences. Later interviews and focus groups demonstrated a high degree of thematic consistency, with discussions largely reinforcing previously identified themes, suggesting thematic saturation. This iterative review and refinement, together with triangulation across focus groups, interviews, and workshop materials, supports the trustworthiness of the findings. An audit trail was maintained throughout the study, including original and coded data, documentation of coding decisions, revisions to the coding framework, and records of team discussions. The analysis emphasized factors that impact biosecurity adoption, including operation or sector specific considerations, and facilitators or incentives to adoption of biosecurity measures. Findings highlighted the need for cross-sector collaboration on biosecurity and the importance of a bottom-up approach to biosecurity that is informed by industry stakeholders.

Workshop data served as complementary participatory evidence: prioritization votes were tallied by criterion within each workshop and summed across the four workshops to identify practices stakeholders viewed as especially feasible, impactful, or urgent. Because participant-added practices could be voted on only where they were proposed, comparisons across practices were restricted to the nine predefined practices that appeared on every ballot, and pooled totals are reported alongside the highest-ranked practices at each workshop (Supplementary Table A.2) because pooling gives more weight to the larger sessions. Vote counts are reported descriptively and were not subjected to statistical testing; and Critical Shifts materials were reviewed thematically for areas for improvement in biosecurity adoption, action steps, and coordination needs. Integrating the two phases, sector and operation-specific perceptions and barriers related to biosecurity adoption identified in the qualitative phase demonstrated the necessity of developing a shared, cross-sector understanding of the current state of biosecurity awareness and adoption and action steps for improving it. Results are presented as a narrative thematic synthesis intended to characterize the range and practical meaning of stakeholder perspectives rather than to estimate the prevalence of views across the industry.

## 3 Results

We organize findings in four parts: how participants framed FMD preparedness as low-priority but high-consequence; the impediments shaping whether biosecurity was seen as practical; the role of incentives, trust, and communication; and the cross-sector workshop priorities that translated these themes into stakeholder-identified actions and critical shifts for improving biosecurity in Colorado’s cattle industry.

### 3.1 FMD preparedness as a low-priority concern

Across sectors, participants described FMD as a distant, low-priority concern. It was hard to motivate action against a disease not currently present in the United States, especially when immediate operational demands crowded out longer-term preparedness. Several participants characterized this as a form of continual triage, meaning that the most urgent daily problem takes precedence over planning for a rare but catastrophic event. This did not mean participants dismissed disease risk. Many distinguished between what they would do during an outbreak and what they could sustain under ordinary conditions, expecting behavior to change quickly once a threat was imminent but doubting that intensive measures could be maintained in the meantime. A recurring suggestion was to frame preparedness around everyday biosecurity rather than FMD alone. Everyday biosecurity could be tied to routine operational concerns and existing educational programs, with FMD serving as an important reminder of why preparedness matters. On the other hand, awareness of the SBS Plan was limited. Most participants had not heard of it, and the few who had generally saw the Plan’s measures as reasonable in principle but hard to implement. This reinforced a broader pattern: stakeholders did not reject preparedness guidance so much as question whether existing tools were simple, accessible, effective, and adaptable enough for real conditions.

### 3.2 Impediments to biosecurity adoption

The most pervasive impediments raised across sectors were time, labor, and cost, which shaped how participants evaluated almost every practice. Planning, documentation, and recordkeeping were seen as necessary but burdensome, particularly for operations with limited labor where biosecurity falls on the producer rather than a dedicated employee. These concerns shaped reactions to a designated biosecurity manager role, which feedlots and larger facilities viewed as feasible but independent producers often saw as an additional responsibility or a potential liability. Participants consistently preferred low-friction resources: one-page guides, checklists, and templates over complex plans or websites. This preference was consistent across sectors, although preferred formats differed by role: producers emphasized one-page checklists and simple guides outlining outbreak actions, while veterinarians emphasized templates they could use to structure biosecurity conversations with producers.

Additionally, several measures were viewed as impractical at the scale of real operations, especially establishing a line of separation (a clearly defined boundary limiting the movement of people, vehicles, and equipment into the area where animals are kept), physical separation, and quarantine. Participants did not reject these in principle but questioned their feasibility given land area, land use types, fencing, shared water sources, multiple entry and exit points, and cattle movement patterns. Impracticality varied by sector: sale barns described receiving animals from many consignors on a single sale day with incomplete disease histories, while feedlots and processing facilities emphasized the constant movement of service providers, transport vehicles, and workers that made vehicle control and disinfection difficult at high throughput.

Participants consistently emphasized that feasibility depends on context, such as operation size, type, geography, and season. Larger operations highlighted challenges of scale in monitoring and traffic control, while smaller ones mentioned limited labor and capacity. Mostly feedlots, processing plants, and some sale barns had existing plans, visitor procedures, or relationships with state and federal veterinarians, whereas extensive cow-calf and stocker operations mentioned struggles with controlled access and continuous monitoring. Altitude, weather, road access, wildlife, and the timing of cattle movement further shaped what was feasible, and some practices were workable as short-term outbreak responses but not as permanent routines – a distinction central to how participants defined “practical” biosecurity. Some also noted that because FMD is highly contagious, individual operation-level measures (such as quarantine, controlled access, visitor hygiene, vehicle cleaning and disinfection, or limiting wildlife contact) might be insufficient on their own, further reducing motivation to invest.

Traceability was among the most challenging measures to sustain, encompassing not only animals but workers, visitors, and vehicles. Barriers included cost, time, privacy concerns, and uneven capacity, with electronic identification associated by some producers with surveillance or loss of autonomy. Ranchers were generally more skeptical than feedlots, processing facilities, and sale barns, which were more familiar with electronic identification and documentation systems; though even these sectors recognized gaps, such as visitor sign-in or vehicle records that would need improvement during an outbreak.

Veterinary access emerged as a major structural constraint. Participants described a shortage of cattle-focused veterinarians in Colorado, particularly in rural areas, who would be central to identifying disease, implementing protocols, and supporting producer decisions during an outbreak. Local veterinarians were generally trusted as familiar and accessible, while state and federal veterinarians were seen as important in emergencies but more distant from local realities. Because of the shortage, participants suggested extension agents and brand inspectors could help fill communication gaps, though not as full substitutes.

### 3.3 Incentives, trust, and communication

Participants, especially producers, emphasized incentives as important for motivating adoption. Economic incentives were salient given the time, labor, and infrastructure many practices require, with financial support, cost-sharing, or market-based mechanisms seen as ways to make investments realistic when benefits are uncertain. However, financial incentives were not viewed as sufficient alone. Participants also identified practical, nonfinancial facilitators, such as simple action-oriented tools, clear guidance, and reduced administrative burden, including one-page summaries distinguishing actions to take before versus during an outbreak, checklists, and templates that veterinarians could use to structure biosecurity conversations with producers. These materials were suggested by participants as potential communication tools rather than evaluated as part of the study.

Trust and autonomy were central to how guidance was evaluated. Producers emphasized voluntary, common-sense practices and resisted mandates, warning that converting recommendations into requirements could erode cooperation. Skepticism toward government-led response was common, including concern that agencies might apply uniform requirements without adequately accounting for differences in operation type, scale, geography, infrastructure, or other practical constraints, while feedlots and processing facilities that routinely interact with the USDA expressed more favorable views. A related concern was fear of reporting, i.e., that producers might delay reporting a suspected case out of uncertainty about consequences, fear of quarantine or depopulation, or reputational risk, which participants saw as a serious threat to early detection. Clearer communication about what happens after reporting, and how producers would be supported, was viewed as essential to trust.

The source delivering information mattered as much as its content. Participants suggested that trust was built through personal relationships, local presence, and practical experience, and producers valued local presence over formal credentials. Local veterinarians, extension agents, and brand inspectors were repeatedly named as trusted messengers, along with producer associations whose reach varied by locality. Because trusted messengers were discussed through open-ended qualitative prompts rather than an individual ranking exercise, frequencies of mentions were not interpreted as prevalence estimates. On format, participants wanted information that was timely, clear, and quick to access: text messaging was preferred for urgent communication and phone calls remained important for older participants, while email was seen as less reliable and social media useful but prone to misinformation. Outdated websites and unclear points of contact were described as sources of distrust, and several participants stressed the need for recurring conversations about FMD, so preparedness does not fade between outbreaks.

Additionally, participants situated individual practices within a broader coordination problem. Because cattle move through multiple sectors with different capacities and regulatory relationships, improving biosecurity was seen to require shared expectations and open communication, not just individual adoption. Brand inspectors and sale barn operators were viewed as well positioned to support information flow during an emergency, and the recurring theme was that practical biosecurity must be defined collaboratively and grounded in operational realities.

### 3.4 Cross-sector workshop priorities

In order to engage participants in a discussion of what constitutes adequate and practical biosecurity, workshop stakeholders were presented with biosecurity practices and asked to identify which practices were most feasible, impactful, and urgent. Participants clearly distinguished among these criteria rather than treating all practices as equally practical. Voting results (dot counts) were summed across the four workshops, and the five predefined practices receiving the most total votes are presented in Table 3, with each count also expressed as a share of all votes cast for that criterion. Across all workshops, the practices receiving the most feasibility votes were hygiene requirements for visitors and vendors (25 votes, 16.8% of feasibility votes cast) and controlling access through restricted entry or defined access points (20 votes, 13.4%). These were viewed as feasible because they are within the control of the operation and most efficient in terms of cost and time to implement. Quarantine of new or returning animals received the most impact votes (23 votes, 14.6%). Among the five practices in Table 3 it was followed by cleaning and disinfection protocols for service providers and transport vehicles (18 votes, 11.4%), although avoiding commingling with animals from other herds, which ranked outside the five most-voted practices overall, received more impact votes (20 votes, 12.7%). These were viewed as more effective in mitigating the risk of disease spread or preventing disease outbreaks altogether. The practices receiving the most urgency votes were quarantining new or returning animals (26 votes, 19.1%) and knowing or asking about disease status of source herds (23 votes, 16.9%), reflecting what participants viewed as most important in the event of an emergency or crisis. Differences between adjacent counts are small relative to the number of votes cast and should be read as a descriptive ordering among these participants rather than as evidence of distinct priority levels. Rankings also varied by workshop location (Supplementary Table A.2): for example, avoiding commingling with animals from other herds received the most impact votes in the Grand Junction and Sterling workshops, and knowing or asking about disease status of source herds received the most feasibility votes in Grand Junction and Fort Collins/Loveland, whereas hygiene requirements for visitors and vendors led on feasibility only in Pueblo, tied with controlling access.

**Table 3:** Dot votes by criterion for the five most-voted predefined biosecurity practices across the four cross-sector workshops.

Biosecurity practice	Impact *n* (%)	Feasibility *n* (%)	Urgency *n* (%)	Total votes
Quarantining new or returning animals	23 (14.6)	17 (11.4)	26 (19.1)	66
Knowing or asking about disease status of source herds	12 (7.6)	19 (12.8)	23 (16.9)	54
Controlling access to the operation (restricted entry/defined access points)	17 (10.8)	20 (13.4)	14 (10.3)	51
Hygiene requirements for visitors/vendors	12 (7.6)	25 (16.8)	5 (3.7)	42
Cleaning and disinfection protocols for service providers and transport vehicles	18 (11.4)	10 (6.7)	13 (9.6)	41
Votes cast, all practices	158	149	136	443

Votes were cast with adhesive dots; each participant received nine dots (three per criterion) and could apply multiple criteria to the same practice. Values are votes summed across the four workshops, with the percentage of all votes cast for that criterion in parentheses (impact = 158, feasibility = 149, urgency = 136; maximum 168 per criterion). All five practices were on the predefined list used at every workshop; practices added by participants at individual workshops are excluded. The complete voting table (Supplementary Table A.1) and the highest-ranked practices at each workshop (Supplementary Table A.2) are presented in the Supplementary material.

The Critical Shifts activity clarified what stakeholders believed needed to change at the system level. Two prominent current-state problems were (i) limited communication and collaboration within and across sectors, and (ii) low producer participation in biosecurity efforts. The desired future state emphasized open communication and collaboration across sectors, with clear first steps and communication protocols. Together, the exercises reframed biosecurity improvement as a practical, cross-sector process. Workshop participants identified specific practices that felt realistic, high-impact, and urgent (such as presented in Table 3), and emphasized the need for shared communication protocols, locally trusted messengers, and industry participation. These findings suggest that improving biosecurity in Colorado will require both low-burden everyday practices and broader coordination structures that can be activated before and during an outbreak.

## 4 Discussion

This study explored how stakeholders across Colorado’s cattle supply chain understand the barriers and incentives shaping biosecurity adoption and FMD preparedness. Although FMD served as the concrete scenario, the findings speak to a broader question: how can preparedness measures be designed, communicated, and coordinated to be feasible before an emergency?

The findings reveal a priority paradox. Participants recognized that an FMD outbreak would be highly consequential and expected behavior to change quickly under imminent threat. Yet FMD’s status as an absent, low-probability, and seemingly distant risk kept it from competing successfully with daily operational pressures. This mirrors previous findings from the United States beef sector. Pudenz et al. (13) found low adoption of strongly recommended pre-outbreak SBS practices, including written, operation-specific biosecurity plans, and attributed this in part to the fact that the benefits of these practices depend on a low-probability event. Similarly, recent survey evidence from Illinois suggests that producers’ perceptions of foreign animal disease preparedness are associated with veterinary engagement, biosecurity evaluations, and willingness to invest in disease prevention (14). These findings indicate that limited adoption should not be interpreted simply as a knowledge deficit or lack of concern. Rather, adoption appears to be constrained by the interaction of motivation, opportunity, and capability. Educational campaigns that only emphasize the severity of FMD are therefore unlikely to be sufficient if recommended actions remain costly, complex, or poorly matched to producers’ operating conditions. Preparedness messaging may be more effective when FMD is framed within everyday biosecurity, where producers can see more immediate relevance and routine benefits.

The workshops show that stakeholders distinguish between measures that are realistic, measures that are high-impact, and measures that are urgent. Practices identified as most feasible, such as visitor and vendor hygiene requirements and controlled access through restricted entry or defined access points, were comparatively low-burden and adaptable. By contrast, practices identified as highly impactful or urgent (quarantining new or returning animals, cleaning and disinfection of vehicles and service providers, and knowing or asking about disease status of source herds) were more directly tied to disease introduction and spread, but were not always viewed as equally easy to implement. This distinction suggests that a single list of recommended practices may be less useful than a tiered approach: low-friction baseline practices that can be adopted broadly, paired with more demanding practices that require support, planning, or sector-specific adaptation.

Feasibility was also highly contextual. Scale, operation type, geography, public-land grazing, wildlife, recreation, transportation, weather, and seasonality all shaped what participants considered practical. Measures such as line-of-separation and quarantine were not rejected in principle but were often judged infeasible given the physical realities of extensive cattle operations, multiple access points, shared fence lines and water sources, public lands, and seasonal cattle movement. This aligns with recent evidence that biosecurity practices differ across beef cattle production types and that prevention strategies should be adapted to the risks and structures of cow-calf, stocker/backgrounder, feedlot, and whole-cycle operations (15). Our findings also support the broader biosecurity literature, which has emphasized that producer perceptions, farm context, and operational constraints shape whether practices are adopted (1, 3, 10). For practice, “realistic biosecurity” should be defined by operation type, available labor and infrastructure, and the relevant risk pathway rather than by uniform expectations. For example, relatively low-burden practices such as visitor/vendor hygiene or defined access points may be feasible for many operations, whereas quarantine, line-of-separation requirements, continuous monitoring, or intensive vehicle disinfection may require substantial adaptation depending on land area, cattle movement, throughput, staffing, and infrastructure.

Some participants questioned whether individual biosecurity measures would be effective against a disease as contagious as FMD, especially when wildlife, insects, airborne transmission, public movement, or shared infrastructure are perceived as beyond producer control. A recent scoping review of farm-level biosecurity measures against FMD similarly concluded that field evidence for many measures remains limited, highlighting the need to communicate uncertainty carefully and avoid overstating the effectiveness of individual practices (6). This does not mean biosecurity is unimportant. Rather, communication should emphasize layered risk reduction, continuity of business, and readiness for response, rather than guaranteed protection.

Trust and autonomy emerged as decisive conditions for adoption of biosecurity measures. Producers’ resistance to top-down mandates and skepticism of distant authorities is consistent with previous work showing that biosecurity recommendations can fail when they are perceived as paternalistic, inspection-oriented, or detached from farm realities (1, 12, 17). In this study, participants did not oppose all guidance. They opposed guidance that seemed unrealistic, poorly communicated, or likely to become punitive. Fear of reporting is a particularly important trust problem because early detection depends on producers feeling comfortable reporting suspicious signs. Preparedness communication should therefore clarify what happens after reporting, who should be contacted, what support is available, and how decisions will be made.

Trusted messengers are not interchangeable. Local veterinarians were trusted because they combine technical expertise with personal relationships and local knowledge, consistent with evidence that the veterinarian-farmer relationship shapes whether biosecurity is discussed and implemented (17). However, the shortage of cattle-focused veterinarians means that veterinary trust cannot be the sole strategy. Extension agents, brand inspectors, sale barns, producer associations, and locally embedded industry leaders can reinforce consistent messages, distribute simple tools, identify information gaps, and direct producers to appropriate contacts. This points toward a distributed communication model, consistent with calls for collaborative, personalized, and sustained biosecurity communication rather than one-way messaging (12).

Coordination across the supply chain may be where the largest gains are possible. Individual operations can adopt some practices on their own, but many important risk pathways involve movement across operations and sectors: new or returning animals, sale barn transactions, feedlot entry, service providers, transport vehicles, veterinary visits, processing facilities, and emergency reporting. Practical coordination could include shared outbreak communication templates, agreed points of contact by sector, consistent language around reporting and movement expectations, and simple checklists tailored to different operation types.

This study has several limitations. It was conducted in a single state and relied on a qualitative, stakeholder-engaged design intended to characterize the range and meaning of perspectives rather than estimate their prevalence across the Colorado or U.S. cattle industry. The sample was self-selected, sector sizes varied, and workshop findings reflect the participants who attended and the structure of the facilitated exercises. The prioritization votes in particular should be interpreted cautiously: dot voting is a visible, sequential procedure in which later voters can see where dots have already been placed, which may have encouraged convergence; pooling counts across workshops gives more weight to the larger sessions; and rankings depend on the composition of the ballot, which is why comparisons were restricted to the practices presented at every workshop. In addition, because FMD was used as the motivating scenario, some responses may reflect assumptions about FMD specifically rather than biosecurity more generally. However, the consistency of themes across focus groups, interviews, and workshops suggests that the barriers identified (time, labor, cost, feasibility, trust, communication, veterinary access, and cross-sector coordination) are broadly relevant to biosecurity planning.

In conclusion, Colorado cattle-industry stakeholders viewed FMD as a distant but high-consequence threat and identified time, labor, and cost as the most pervasive barriers to voluntary biosecurity adoption, compounded by operation-specific constraints involving scale, geography, seasonality, traceability, and veterinary access. Taken together, the findings suggest a practical sequence for strengthening preparedness. First, reducing time and administrative burden through concise, action-oriented tools and clearly defined expectations may make routine biosecurity easier to adopt. Second, financial support or cost-sharing may be necessary where implementation requires additional labor, infrastructure, or equipment. Third, guidance should be delivered through trusted local messengers and should clearly explain reporting procedures, expected consequences, and available support. Finally, cross-sector communication protocols and relationships should be established before an emergency occurs. Within this framework, visitor/vendor hygiene and controlled access provide relatively feasible starting points, while quarantine of new or returning animals, cleaning and disinfection of service providers and transport vehicles, and source-herd disease information represent especially high-impact or urgent preparedness priorities.

## Data Availability

The raw data supporting the conclusions of this article will be made available by the authors, without undue reservation.
