# Supplementary material for: Stakeholder perspectives on biosecurity adoption in the Colorado cattle supply chain: lessons from foot-and-mouth disease preparedness
Source: Front Vet Sci. 2026 Oct 2;13:1937939. doi: 10.3389/fvets.2026.1937939 (PMC13672825; doi:10.3389/fvets.2026.1937939)
Supplement: Supplementary file 1 [file Data_Sheet_1.PDF]

Supplementary Material

FOCUS GROUPS | CVMBS FMD Project

Introduction Questions [10 minutes]

1. Let's start with some introductions. Please share your first name and your background in the cattle industry.
2. Now, let's talk broadly about biosecurity in the cattle industry. What role do you think [sector] play in biosecurity?

Factors Impacting Biosecurity [25 minutes)

Next, we are going to talk about different factors or challenges that impact biosecurity in the cattle industry.

3. First, we'll start with factors that impact biosecurity as they relate to you and your role – what factors or challenges related to biosecurity impact you as a [role type]?
4. Now, let's talk about other people in the industry that you deal with, people like [examples]. What challenges related to biosecurity come into play when dealing with others in the supply chain?
5. Now let's talk a little bit about the region you work in. Are there any biosecurity factors or challenges that are specific to your region?
    - Prompts: are there seasonal factors that impact biosecurity? Are there factors biosecurity factors or challenges related to transportation?

SBSP Feedback

6. How familiar are you with the Secure Beef Supply Plan **OR** Secure Milk Supply Plan?

   Now we are going to talk about some of the concepts that the SBSP says [sector] should be ready to implement prior to an FMD outbreak in the U.S. We'd like to hear your thoughts about how practical they would be to implement.

7. **Designating a Biosecurity Manager**
    a. Every feedlot should appoint a Biosecurity Manager who is responsible for developing the biosecurity plan *before* any outbreak occurs.
    b. Plan should include:
        i. Outline scope of the operation
        ii. Documentation forms: training / signatures

iii. Procedures and signage used on feedlot
iv. Ensure protocols are clearly communicated

8. Pre-Movement Isolation Period

a. Animals from FMD control area should not be introduced to the feedlot for at least 7 days before moving animals to another site with susceptible animals

9. Line of Separation

a. Biosecurity plan should include an outer boundary around or within premises that restricts movement of pathogens into areas with susceptible animals

10. Cleaning & Disinfection Station (C&D Station)

a. Clearly marked & fully equipped to remove visible contamination & disinfect vehicles, equipment, and other things crossing line of separation.
b. Trained personnel,
c. Runoff to be managed according to state/local regulations to prevent contamination of waterways, etc.

Closing Questions

11. Who are the people or organizations you get biosecurity information from?
    - Who do you trust to provide accurate and useful information related to biosecurity?
    - How would you work with them if an FMD outbreak were to occur?

12. [***IF not covered in activity***] What do you as [role type] need to improve your biosecurity?
    - What additional tools or guidance would be helpful for you?

13. Is there anything else that you would like to share related to biosecurity or foot and mouth disease that we have not discussed yet today?

WORKSHOP ACTIVITY 1 | FMD PARTICIPANT GUIDE

Discussion 1: Priority Setting

Step 1: Brainstorm biosecurity practices

- Review list of biosecurity practices
- Brainstorm missing biosecurity practices- *what else can you think of?*
    - Create new post its with new practices

Step 2: Vote

- Each person has 3 different colors of sticky dots, 3 of each color (9 total).
- Each color represents different traits:
    - **Impact = Yellow**: Which practices do you believe would have the most impact to improve biosecurity / reduce disease risk?
    - **Feasibility = Green**: Which practices feel the most realistic to implement, given time, cost, labor, and day-to-day realities?
    - **Urgency = Red**: Which practices feel the most pressing right now?
- Each person gets to "vote" with their sticky dots.
- *Each person makes their own votes.*
- You can place different color dots on the same practice (for example, a practice can be both feasible & urgent). <u>Please place them on the sticky note</u>.

Step 3: Discuss & Capture Reasoning

After you vote, talk through *why* you placed dots where you did. <u>These explanations are just as important as the votes themselves.</u>

<u>Turn to back for discussion prompts. Talk through these questions together</u> →

Discussion Prompts

<u>Feasibility</u>

- What makes this practice feel more doable compared to others?
- What are attributes that make something feasible?
    - *Things to think about- cost, time, labor, knowledge- what else?*
- Feasible for whom? How does feasibility vary by operation type?

<u>Impact</u>

- Why would you say this practice is more impactful than others on the list?
- Think about timeline- are the impacts immediate or long-term?
- Is this impactful day-to-day? Or is the impact only visible in the case of a big event?
- If this practice requires significant investment, does the impact justify it?

<u>Urgent</u>

- What makes this feel urgent right now?
    - *Is it urgent because of disease risk? Or because of regulation, market pressure, recent events- other?*
- Is this addressing a frequent, everyday problem or a rare but catastrophic one?
- Is something urgent in one sector of the industry but not another? Why?

<u>Characteristics of Practical & Adequate Everyday Biosecurity</u>

- Everyday biosecurity is biosecurity that is …
- Biosecurity stops being practical when …
- What can we do to move the needle to improve everyday biosecurity?
    - *Think of incentives to motivate adoption*
    - *Think of barriers that keep people from adopting*

Biosecurity Practices

- Knowing / asking about disease status of source herds
- Quarantining new / returning animals
- Testing incoming animals for diseases (beyond regulatory requirements)
- Vaccination status of animals (source herds & home herd)
- Avoiding commingling with animals from other herds
- Controlling access to the operation (restricted entry / defined access points)
- Cleaning & disinfection protocols for service providers & transport vehicles
- Hygiene requirements for visitors/vendors
- Controlling wildlife on operation (e.g., wildlife access, insect control)

WORKSHOP ACTIVITY 2 | CRITICAL SHIFTS

The purpose of this activity is to identify and reach a consensus related to what areas the industry should collectively prioritize to move the work of improved biosecurity forward.

The goal of this activity is to **identify potential action steps within and across sectors** to strengthen biosecurity and outbreak preparedness.

OVERVIEW

Critical shifts are specific "problem or priority spaces" that stakeholders agree to focus on designing solutions for. The task of defining and prioritizing critical shifts helps us to identify how best to bridge the current reality related to biosecurity in the cattle industry to a shared future goal of stronger biosecurity.

Each Critical Shift has two parts – a short description of a specific aspect of biosecurity that is NOT currently working – and a description of the ideal future state of biosecurity in the cattle industry. When drafting critical shifts, it is important to:

- Remember to use neutral language - no blame or judgement.
- Avoid including any potential solutions in your description

Examples of Critical Shifts in the Cattle Industry

Current State	Future State
Producers experience a lack of access to vets in rural areas.	All producers have adequate access to vets, including in rural areas.
Certain biosecurity strategies are difficult to implement for producers that graze on public lands.	There are biosecurity strategies that are adaptable for producers that graze on public lands.
There is a lack of communication and information available to various sectors related to biosecurity.	All sectors have adequate information and communication networks related to biosecurity.

Small Group Discussion: Part 1 [15 minutes]

1. In your small groups, take several minutes to brainstorm possible critical shifts (current state/ideal future state) related to biosecurity in the cattle industry. Use your large sticky note to list out all of your critical shifts. *REMEMBER – do not assign blame in current state; do not include solutions in future state***.**

2. In your group, choose your <u>three top critical shifts</u>. If you have several similar critical shifts, group them together. Ask yourselves the below questions to help identify which are your top priorities:

   - What critical shift will advance goal of better biosecurity most powerfully?
   - What critical shift, if we achieved it first, would enable other shifts to happen more easily or more powerfully?
   - What critical shift would NOT happen without a collaborative effort across industry sectors?
   - What critical shift would generate broad energy and buy-in form stakeholders and the community?

3. Be prepared to report back to the larger group: share out what your top three critical shifts are. No need to go into great detail now.

Large Group Discussion [25 minutes]

Small Group Discussion: Part 2 [15 minutes]

4. In your new small groups, focus on your critical shift and discuss the following questions:

   - What are realistic steps that could be taken toward addressing this shift in the next year?
   - Who would need to be involved for this shift to move forward?
   - What is the role each of the sectors play in this critical shift?

5. Be prepared to report back to the larger group: share out what next steps you have identified, how you see as needed to be involved in making change happen, which sectors need to be involved?

Large Group Discussion [15 minutes]

Table A.1 Prioritization Activity: All Votes

Biosecurity Strategy	Impact	Feasible	Urgent	Total votes
Quarantining new / returning animals	23	17	26	**66**
Knowing / asking about disease status of source herds	12	19	23	**54**
Controlling access to the operation (restricted entry / defined access points)	17	20	14	**51**
Hygiene requirements for visitors/vendors	12	25	5	**42**
Cleaning & disinfection protocols for service providers & transport vehicles	18	10	13	**41**
Avoiding commingling with animals from other herds	20	9	6	**35**
Testing incoming animals for diseases (beyond regulatory requirements)	18	7	7	**32**
Vaccination status of animals (source herds & home herd)	7	12	11	**30**
Controlling wildlife on operation (e.g., wildlife access, insect control)	13	3	3	**19**
Record keeping	6	2	7	**15**
Communication (between buyer/seller)	2	2	5	**9**
Cleanliness of transport vehicles		6	2	**8**
Public Awareness of Biosecurity		2	4	**6**
Educate "scare" young producers/4-H	3	1	2	**6**
Insect control	3	1	2	**6**
Restricted entry to the operation	2	3	1	**6**
Sharing of equipment of vehicles w/other operations		3	2	**5**
Preventing animal access to fee storage		3	1	**4**
Managing visitor access & contact w/other animals	1	2		**3**
Having a SBSP			1	**1**
Informing the community/education			1	**1**
Parasiticides/other prevention		1		**1**
Post Biosecurity Area/Awareness		1		**1**
Render pickup truck/sick animal pickup	1			**1**
Controlling Access on Public Lands				**0**
Traceability				**0**
**Votes cast, all practices**	**158**	**149**	**136**	**443**

Note: Votes were cast with adhesive dots; each participant received nine dots (three per criterion). The first nine practices were on the predefined list presented at all four workshops; the remaining practices were added by participants and could receive votes only at the workshop(s) at which they were proposed, so their totals are not

directly comparable with those of the predefined practices. Attendance was 10 (Sterling), 16 (Pueblo), 17 (Fort Collins/Loveland), and 13 (Grand Junction); the maximum possible number of votes per criterion across the four workshops was 168.

Table A.2 Highest-ranked practice at each workshop, by criterion

Workshop	n	Feasibility	Impact	Urgency
Sterling (Jan 27)	10	Cleanliness of transport vehicles*	Quarantining new / returning animals; Avoiding commingling with animals from other herds (tie)	Knowing / asking about disease status of source herds
Pueblo (Feb 9)	16	Hygiene requirements for visitors/vendors; Controlling access to the operation (tie)	Testing incoming animals for diseases (beyond regulatory requirements)	Quarantining new / returning animals
Fort Collins/Loveland (Feb 16)	17	Knowing / asking about disease status of source herds	Controlling access to the operation (restricted entry / defined access points)	Controlling access to the operation (restricted entry / defined access points)
Grand Junction (Mar 11)	13	Knowing / asking about disease status of source herds	Avoiding commingling with animals from other herds	Knowing / asking about disease status of source herds; Vaccination status of animals; Cleaning & disinfection protocols for service providers & transport vehicles (three-way tie)
All workshops (pooled)	56	Hygiene requirements for visitors/vendors	Quarantining new / returning animals	Quarantining new / returning animals

Note: n = number of participants at each workshop. Where more than one practice is listed, the practices received the same number of votes at that workshop. *Participant-added practice, votable only at the workshop where it was proposed; all other practices listed were on the predefined list used at every workshop.
